# Nitrites and nitrates from additives and natural sources and risk of cardiovascular outcomes

**DOI:** 10.1093/eurpub/ckac129.491

**Published:** 2022-10-25

**Authors:** B Srour, E Chazelas, C Debras, N Druesne-Pecollo, C Agaesse, F Szabo de Edelenyi, L Sellem, E Kesse-Guyot, M Deschasaux-Tanguy, M Touvier

**Affiliations:** EREN, Inserm, Inrae, Cnam, USPN, INSERM, Bobigny, France

## Abstract

Nitrates and nitrites are used as food additives in processed meats. They are also commonly ingested from water and several foods. Evidence suggests a beneficial role of dietary nitrites and nitrates in lowering blood pressure. However, associations between exposure to nitrites and nitrates from natural sources and food additives, separately, and risks of hypertension and cardiovascular disease (CVD) have not been investigated. We aimed to study these associations in the French population based prospective cohort NutriNet-Santé. Overall, 104,817 adults were included. Associations between exposure to nitrites and nitrates (evaluated using repeated dietary records, linked to a food composition database accounting for commercial brands of industrial products) and risks of hypertension and cardiovascular disease were assessed using multivariable Cox proportional hazard models. During follow-up, 3810 incident cases of hypertension were ascertained, and 2075 cases of CVD, 1004 of cerebrovascular diseases and 1079 or coronary heart diseases were diagnosed. Participants with higher exposure to nitrites from food additives and specifically those highly exposed to sodium nitrite (e250) had a higher hypertension risk compared with those who are not exposed to nitrites from food additives (HR = 1.19 (95% CI 1.08-1.31), P = 0.002, and 1.19 (95% CI 1.07-1.31), P = 0.002, P < 0.001), respectively). There was no evidence for an association between total nitrites or nitrites from natural sources, or dietary nitrates with hypertension risk (all P-values>0.3). There was no evidence for associations between dietary nitrites, or nitrates with risks of cardiovascular, cerebrovascular or coronary heart diseases (all P-values>0.2). In conclusion, we found that higher exposure to nitrites from food additives was associated with higher risk of hypertension. Our results do not support a potential protective association between dietary nitrites or nitrates and cardiovascular outcomes.

**Key messages:**

• These results provide additional evidence in the context of current discussions about updating regulations on the use of nitrites as food additives.

• Our findings do not support any protective impact of nitrites and nitrates on cardiovascular health.

